# A functional investigation of the suppression of CpG and UpA dinucleotide frequencies in plant RNA virus genomes

**DOI:** 10.1038/s41598-019-54853-0

**Published:** 2019-12-04

**Authors:** Ahmad Ibrahim, Jelke Fros, Andre Bertran, Ferdyansyah Sechan, Valerie Odon, Leslie Torrance, Richard Kormelink, Peter Simmonds

**Affiliations:** 1 Nuffield Department of Medicine, Peter Medawar Building for Pathogen Research, University of Oxford, Oxford, OX1 3SY UK; 2 Laboratory of Virology, Wageningen University, Droevendaalsesteeg 1, 6708 PB Wageningen, The Netherlands; 3 The James Hutton Institute, Invergowrie, Dundee, DD2 5DA UK

**Keywords:** Molecular evolution, Virus-host interactions, Innate immunity

## Abstract

Frequencies of CpG and UpA dinucleotides in most plant RNA virus genomes show degrees of suppression comparable to those of vertebrate RNA viruses. While pathways that target CpG and UpAs in HIV-1 and echovirus 7 genomes and restrict their replication have been partly characterised, whether an analogous process drives dinucleotide underrepresentation in plant viruses remains undetermined. We examined replication phenotypes of compositionally modified mutants of potato virus Y (PVY) in which CpG or UpA frequencies were maximised in non-structural genes (including helicase and polymerase encoding domains) while retaining protein coding. PYV mutants with increased CpG dinucleotide frequencies showed a dose-dependent reduction in systemic spread and pathogenicity and up to 1000-fold attenuated replication kinetics in distal sites on agroinfiltration of tobacco plants (*Nicotiana benthamiana*). Even more extraordinarily, comparably modified UpA-high mutants displayed no pathology and over a million-fold reduction in replication. Tobacco plants with knockdown of RDP6 displayed similar attenuation of CpG- and UpA-high mutants suggesting that restriction occurred independently of the plant siRNA antiviral responses. Despite the evolutionary gulf between plant and vertebrate genomes and encoded antiviral strategies, these findings point towards the existence of novel virus restriction pathways in plants functionally analogous to innate defence components in vertebrate cells.

## Introduction

There is increasing evidence that many phenotypic properties of a virus and aspects of its interaction with hosts are determined by structural and compositional attributes of its genome. Single stranded RNA virus genomes may be internally base-paired, creating often extensive duplex or higher order structures that interact with replication proteins or translation factors^1,2^. Viruses may also display compositional abnormalities, most notably, suppression of the frequencies of CpG and UpA dinucleotides that have, in mammalian RNA viruses and retroviruses, been shown to influence replication^3–6^. In part, these biases in RNA viruses may be influenced by the genomic composition of the hosts they infect, potentially mirroring the suppression of CpG and TpA dinucleotides in vertebrate and plant DNA genomes^7–9^. In these latter groups, suppression of CpG dinucleotides originates from a mechanism in which the cytosine of the CpG dinucleotides is targeted for methylation rendering it susceptible for mutation to thymine by deamination^10^. Genomes of methylation-heavy organisms such as vertebrates and higher plants show extensive under-representation of CpG dinucleotides^11,12^. It is estimated that 70–80% of cytosines in CpG motifs are methylated in mammalian genomes^13^ while CpG methylation in plants is much less extensive (24%)^14^ and largely limited to transposons and other repetitive DNA elements^15^. Plant genome methylation also occurs in a wider range of contexts, with 1.5% of cytosines methylated in CHH motifs (where H = non-G *ie*. A, C or T) and around 6.7% in CHG sites^14^. Methylation plays essential regulatory roles in gene expression with functions ranging from epigenetic control^16^ and anti-viral defence via transcriptional (transposons) gene silencing. The latter is mediated by the RNAi pathway and requires the biogenesis and amplification of 24 nucleotide (nt)-sized small-interfering (si)-RNAs by DCL3 and RNA-dependent RNA polymerase 2 (RDR2), respectively^17^. Less is understood about UpA dinucleotides suppression. However, RNA degrading nucleases involved in mRNA turnover may target UpA dinucleotides and U + A rich sequences in mRNA for degradation and UpA frequencies may be regulated for this reason^7,18^.

In general, the genomic composition of eukaryotic RNA viruses often recapitulates the compositional characteristics of host mRNA and, in the case of vertebrate and plant viruses, may reproduce the suppressed frequencies of both CpG and UpA dinucleotides of their hosts^19^. However, while it could be conceived that suppression of UpA may occur in response to the same cellular factors that govern the stability of cellular mRNAs^18^, what mediates or drives the suppression of CpGs in RNA virus genomes is unclear given that CpG-associated methylation and loss of CpGs is a specifically DNA-based mechanism occurring in the nucleus. In mammalian RNA viruses, artificially increasing CpG and UpA frequencies in a range of mammalian RNA virus genomes have a severe attenuating effect on replication of echovirus 7 (E7) that is independent of interferon-mediated cellular responses to virus infection, stress response pathways and apoptosis^3,5^. Recently, zinc finger antiviral protein (ZAP) was shown to directly bind high CpG RNA sequences of HIV-1 and restricted its replication^4^. Our recent investigation of restriction mechanisms found no evidence for translational arrest^20^ or RNA exonuclease-mediated digestion or cleavage of high CpG mutants of E7. We have recently obtained evidence that, in addition to CpG dinucleotides, UpA dinucleotides may be similarly targeted by ZAP and moreover, by RNAseL, a site-specific RNA nuclease may contribute to the attenuation of high CpG mutants of E7^21^.

In contrast to mammalian viruses, there is currently no functional data on effects of similar manipulation of CpG or UpA frequencies on the replication of plant viruses. Their extensive suppression argues that they may be under similar restrictive pressures to enable efficient replication. To address this, we used the PVY-N605 strain of potato virus Y (PVY)^22^ as a model virus for mutagenesis and phenotypic characterization using the previously developed infectious clone^23^. PVY is a member of the *Potyviridae* family and particles consist of a non-enveloped filamentous structure containing a positive-sense single-stranded genomic RNA^24^, of approximately 9.7 Kb in length. The genomic RNA contains an open reading frame (ORF) encoding viral structural and replication proteins, and a short overlapping ORF encoding pretty interesting potyviral protein^24^. The PYV coding region is flanked by short 5′ and 3′ untranslated regions (UTRs), and with a viral protein genome-linked (VPg) covalently attached to its 5′ end and a 3′ poly(A) tail^25,26^. An *Agrobacterium tumefaciens* mediated delivery method for the infectious clone of PVY^NTN^ was used to investigate the effect of elevated dinucleotide frequencies on virus replication and systemic spread in *Nicotiana benthamiana*.

## Results

### Suppression of CpG and UpA frequencies in genomes of plants and plant viruses

**Figure 1:**
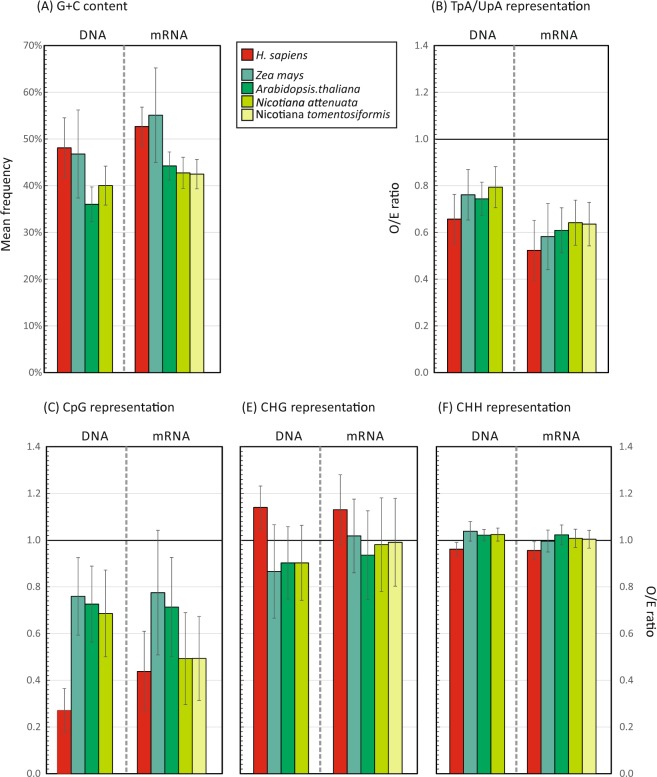
G + C content and frequencies of CpG, TpA/UpA, CHG and CHH in plant genomes. G + C content and frequencies of TpA/UpA and CpG dinucleotides and CHG and CHH motifs in genomic DNA (fragment sizes 5000 bps) and coding regions of mRNAs (>450 bases) of three different plant species and in the human genome. Dinucleotide frequencies were expressed as ratios of observed (O) numbers divided by the expected (E) numbers based on frequencies of their constituent bases or combinations of bases. Expected frequencies of CHG and CHH motifs were calculated by references to frequencies of their component dinucleotides (see Methods). For each, an O/E ratio of 1.0 (dotted line) is the expected frequency. Bar heights show mean values; error bars show ±1 standard deviation.

To quantify the degree of suppression of CpG and UpA dinucleotides in plant viruses and how this compares with host plant genomic composition, we first analysed CpG, CHG, CHH and TpA frequencies in genomic DNA and in the subset of coding sequences expressed as mRNAs in several example plants. These included the annotated genomes of *Nicotiana* species, *N. attenuata* and *N. tomentosiformis* (similar to the experimental plant used in the study, *N. benthamiana* whose genome is currently incompletely annotated), *Arabidopsis thaliana* and *Zea mays* (a monocotyledon), along with corresponding sequence datasets from the human genome (Fig. 1). Suppressed frequencies of CpG were consistently observed in genomic DNA and mRNA of all plant species analysed, although to a lesser extent than observed in human DNA (Fig. 1C) and other mammals^11^. There was also substantial suppression of UpA in plant mRNA sequences that was consistently greater than the corresponding of TpA in genomic DNA (Fig. 1B). There was a much lower degree (8%) of suppression of CHG sites in plant genomic DNA sequences and largely absent in the corresponding mRNA sequences (Fig. 1E). Finally, there was no evidence for any consistent suppression of CHH trinucleotides in the plant sequence datasets, consistent with the very low degree of methylation of this motif in plant genomes.

**Figure 2:**
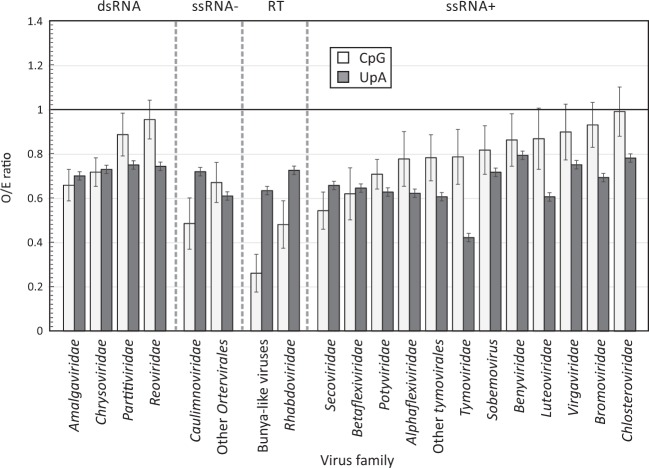
Comparison of CpG and UpA compositions in different plant RNA virus families. O/E ratios of CpG and UpA dinucleotides in 2612 individual protein or polyprotein genes (>450 bases) derived from 1193 plant virus genomes in the ICTV Virus Metadata Resource (https://talk.ictvonline.org/taxonomy/vmr/) divided into separate Baltimore groups and families. These comprise dsRNA RNA viruses (Baltimore group 3), negative-stranded RNA viruses (ssRNA−; Group 5), reverse transcribing viruses (RT; Group 7) and plus-stranded RNA viruses (ssRNA+; Group 4). Bar heights show mean values; error bars show ± 1 standard deviation.

The extent to which plant RNA viruses mimic the compositional features of their host was analysed using exemplar genomic sequences of each major plant virus family (Fig. 2). Suppression of UpA was observed in all virus families and groups (mean O/E ratio 0.68; range 0.42–0.79), and was comparable to those of plant cellular mRNAs (mean 0.62, range 0.58–0.64 for the four plant species analysed). CpG suppression was however, more variable. CpG was substantially suppressed in ssRNA- *Rhabdoviridae*, the ambisense bunyaviruses and reverse transcribing viruses with O/E ratios ranging from 0.26–0.67. There was however, much more variable suppression in the ssRNA + viruses (O/E range 0.54–1.00) and dsRNA viruses (0.66–0.95). For many families in these latter groups, CpG frequencies were substantially higher than those of the analysed plant species (0.45–0.79). For viruses showing suppression of CpG (*Secoviridae*, *Potyviridae, Tymovirales*, luteo/tombus/sobemoviruses and ambi-/minus strand RNA viruses), frequencies were associated with their G + C content (Fig. S1A, Suppl. Data; R^2^ = 0.317; *p* < 0.0001), a relationship that closely recapitulates that observed in vertebrate RNA viruses^11^. There were however, a number of virus groups, including the dsRNA viruses (*Reoviridae, Chrysoviridae, Partitiviridae*) and several virus groups of + strand RNA viruses (*Beniviridae, Closteroviridae, Virgaviridae* and *Bromoviridae*) that did not display this relationship (Fig. S1B; Suppl. Data). This difference potentially relates to structural factors, such as exposure of virus genomic RNA to the cytoplasm. Finally, in common with plant mRNA sequences, there was no suppression of CHG or CHH frequencies in any virus family/group (Fig. S2; Suppl. Data).

#### Development of a plant virus model

**Figure 3:**
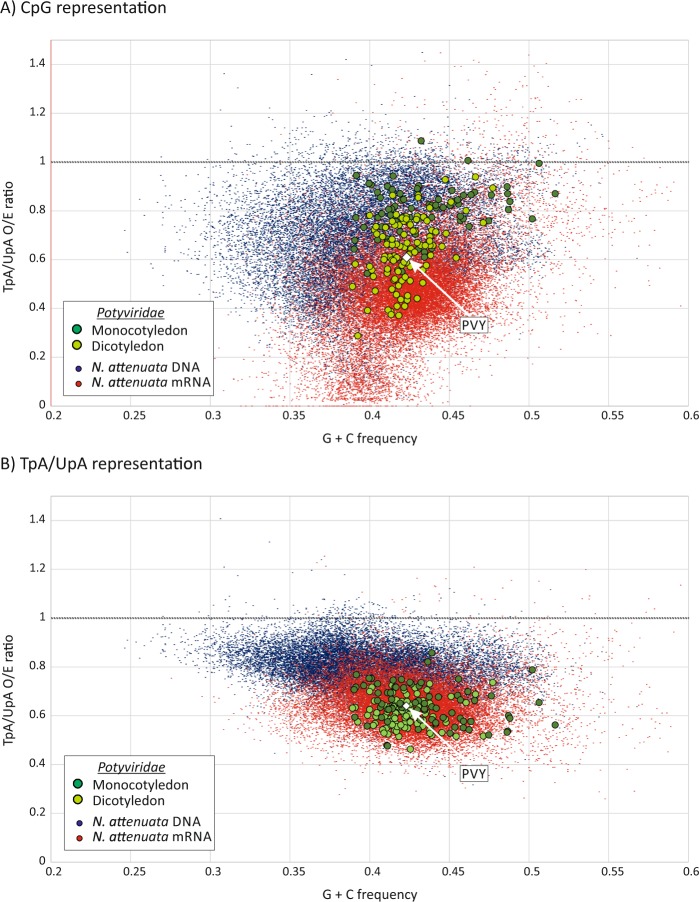
CpG and TpA/UpA dinucleotide representation in *N. attenuata* and potyviruses. Frequencies of (**A**) CpG and **(B**) TpA/UpA dinucleotides in *N. attenuata* genomic DNA (5000 bp fragments), coding regions of mRNAs (>450 bases) and polyprotein genes of each potyvirus species (>450 bases). Potyviruses have been divided into those infecting dicotyledons (light green) and monocotyledons (dark green). The composition of the PYV strain used in the current study is highlighted (white diamond symbol). The O/E ratio of 1.0 is drawn as a heavier horizontal line.

The current analysis included polyprotein sequences of members of the *Potyviridae* family. These show a level of UpA suppression typical of plant RNA viruses (mean O/E value 0.63 ± 0.07 among the 202 genome sequences analysed, and suppression of CpG frequencies (0.71 ± 0.07) (Fig. 2). Compositionally, potyviruses closely matched the mRNA sequences of plants, both in terms of dinucleotide suppression and G + C content (Figs. 1 and 3). Furthermore, the greater degree of CpG suppression observed in the dicotyledons *A. thaliana* and *Nicotiana* spp. compared to the monocotyledon, *Z. mays* (Fig. 1) was recapitulated among potyviruses (Fig. 3A). Those infecting dicotyledons showed a mean CpG O/E of 0.63 (±0.13) that was significantly lower than those infecting monocotyledons (mean 0.80, ±0.10; *p* < 10^−9^ by Kruskal-Wallace non-parametric test). UpA frequencies were however comparable both between mono- and cotyledon mRNA sequences (Fig. 1) and potyviruses infecting these hosts (Fig. 3B). The differences in G + C contents genomic DNA of dicotyledons (36–40%) and monocotyledons (47%) and their corresponding mRNA sequences (43–44%, compared to 55%) was also not reflected in genome compositions of potyviruses infecting these two hosts (42.3% and 43.5% respectively). CHG and CHH motif frequencies in PYV and *N. attenuata* were comparable (Fig. S3; Suppl. Data).

For the selected experimental system, there was a close compositional match between PVY (indicated as a white diamond in Fig. 3A,B) with mRNA sequences of *N. attenuata* and *N. tomentosiformis* (Figs. 1 and 3). PYV is a necrotic strain but degrees of CpG and UpA suppression between ordinary and necrotic (PVY^NTN^ and PVY^N-Wi^) strains were comparable (Fig. S4; Suppl. Data).

**Figure 4:**
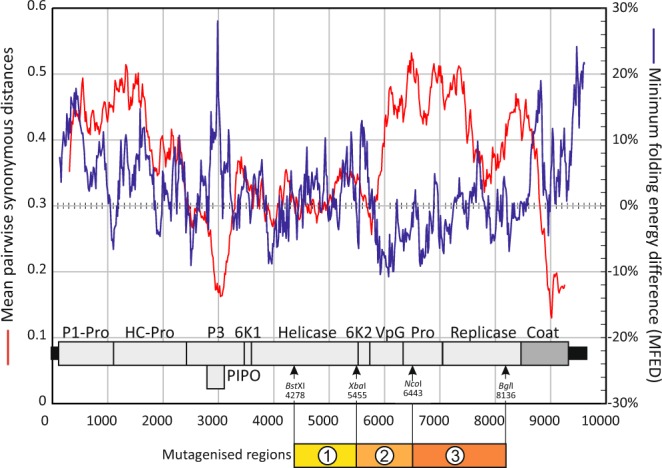
Synonymous variability and RNA structure predictions in the PYV genome. Scan of synonymous variability (red line; left hand axis scale) between aligned PYV sequences (O- and NTN-groups – see Methods for sequence listing) to identifiy regions of suppressed variability indicative of overlapping reading frames and structured RNA elements. A plot of MFED (blue line; right hand y-xaxis scale) has been superimposed to identify areas of thermodynamiclly favoured base pairing. The position of the mutated regions are shown underneath the genome diagram (see Fig. S5, Suppl. Data). Gene abbreviations: P1-Pro: protein required for genome replication. HC-Pro: helper component-protease. PIPO: protein required for viral replication and a recently recognized protein implicated in cell to cell movement. 6k1: exact function unknown. 6K2: membrane anchoring protein. VPg: genome-linked protein. Pro: nuclear inclusion proteinase. Coat: capsid protein.

To investigate whether CpG and UpA dinucleotide frequencies influenced PVY replication and pathology, six mutant viruses were designed in which their frequencies were modified (Table 1). Genomic regions that showed a suppression of synonymous site variability and areas of predicted RNA structure (Fig. 4) were avoided for mutagenesis as this may disrupt underlying replication elements and overlapping alternative open reading frames. These included the site of the PIPO CDS and at the 3′end of the coding sequence. Elevated minimum folding energy differences (MFEDs) provided evidence of areas of RNA structure in the region encoding PIPO and at the 3′ genome end. However, regions between the helicase and replicase encoding genes showed MFED values of around zero and little suppression of synonymous variability. A genomic fragment extending between restriction sites *Bst*XI (position 6443) and *Hpa*I (position 8354) of the N19N21 clone of PVY (Figs. 4 and S5, S6; Suppl. Data) genome was therefore selected and sub-cloned for further manipulation.

**Table 1:** CpG and UpA dinucleotide composition of PVY and mutated regions used in this study.

Region	Label	Sequence Composition	Total Nucleotide	Total CpG (Change)	Total UpA (Change)	Ratio CpG	Ratio UpA
**Full PVY**	**WT PVY**	**Native**	**9701**	**248 (−)**	**540 (−)**	**0.613**	**0.647**
**Region 3**	WT	**Native**	**1699**	**37 (−)**	**94 (−)**	**0.535**	**0.636**
Region 3	R3-CpGH	CpG high	1699	162 (+125)	90 (−4)	2.193	0.635
Region 3	R3-UpAH	UpA high	1699	37 (0)	192 (+98)	0.535	1.299
Region 3	R3-CDLR	CDLR	1699	37 (0)	94 (0)	0.535	0.636
**Combined Regions 1, 2, & 3**	WT	**Native**	**4066**	**82 (−)**	**241 (−)**	**0.524**	**0.655**
Combined Regions 1, 2, & 3	R123-CpGH	CpG high	4066	446 (+364)	236 (−5)	2.150	0.7855
Combined Regions 1, 2, & 3	R123-UpAH	UpA high	4066	84 (+2)	523 (+283)	0.626	1.305
Combined Regions 1, 2, & 3	R123-CDLR	CDLR	4066	83 (+1)	241 (0)	0.532	0.654

**Figure 5:**
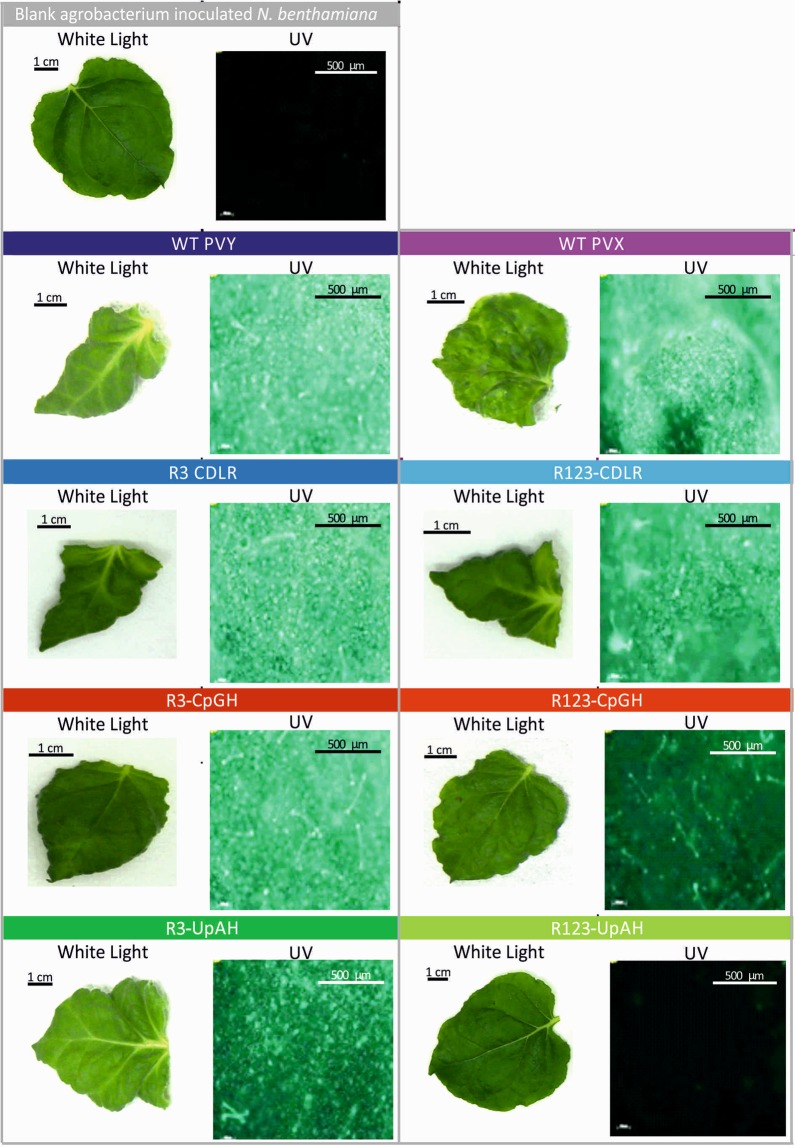
Morphology and virus detection in *N. benthamiana* leaves after infection with PVX, WT PVY and PVY mutants. Phenotype comparison and green fluorescence protein (GFP) detection in the topmost third leaf of *N. benthamiana* agroinoculated (OD_600_ 0.4) of PVY and compositionally altered mutants of PVY, along with PVX. Leaves were harvested at 16 dpi. Replicating PYV was visualized by fluorescence microscopy. The scale bar is 100 µm.

PVY mutants were constructed with modifications to region 3 (R3; positions 6443–8136 nt) representing 18% of the genome (Table 1; Fig. 5; Suppl. Data). These included R3-CpGH (maximised CpGs), R3-UpAH (maximised UpAs) and R3-CDLR (randomly permuted control (retaining native mono- and dinucleotide frequencies and coding). CDLR mutagenesis would destroy any replication elements or alternative reading frames undetected on the genome scan that might otherwise influence PYV replication. A corresponding set of region 123 mutants contained an extended region of mutagenesis between positions 4278–8136 (42% of the genome) were also constructed. For visual monitoring of replication *in planta*, all PVY constructs contained GFP (Fig. S5, Suppl. Data), while a potexvirus PVX-GFP^27^ construct (Fig. S5, Suppl. Data), was used as control for agroinoculation.

### Effects of compositional modification on replication

To first observe the systemic spread and phenotype of the WT PVY together with the six PVY mutants, *N. benthamiana* plants were agroinoculated with *A. tumefaciens* containing binary constructs of the infectious clones and visually monitored for disease symptomatology and GFP expression. Plants were observed for 16 days post inoculation (dpi) over which time symptoms of necrosis, stunted growth, and leaf curling developed in plants infected with WT PVY and PVX (Fig. 5). Viral systemic spread from the agroinoculation site through the plant vascular system, to the upper leaves was observed (Fig. S7; Suppl. Data). Green fluorescence was detected in leaves harvested at 7 dpi, 10 dpi, and 16 dpi in PVX, WT PVY, R123-CDLR, R3-CDLR, R3-CpGH, and R3-UpAH agroinoculated plants (Fig. 5).

**Figure 6:**
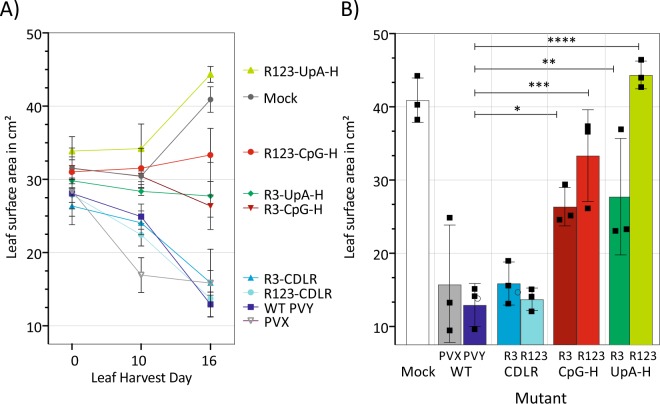
Quantification of pathology changes in tobacco plants infected with PYV. (**A**) Time course for the decline in mean leaf area of the four top most leaves of PVY mutants agroinoculated (OD_600_ 0.4) *N. benthamiana* at 7, 10, and 16 dpi. (**B)** Comparison of the uppermost four leaves surface area harvested from three plants at 16 dpi. Bar heights show mean values; error bars show standard deviations, square data-points indicate values for separate biological replicates. Areas were compared using One-way ANOVA Tukey multiple comparisons test; significance values of differences from the WT virus are indicated above bars; *p = 0.0215; **p = 0.0099; ***p = 0.0004, ****p < 0.0001.

To quantify stunted growth, the upper-most four (systemically infected) leaves of *N. benthamiana* were harvested at 07, 10, and 16 dpi and their leaf surface areas measured. Plants agroinoculated with the PVX, WT PVY and the CDLR mutants showed a substantial and progressive decrease in leaf size, with the three replicate WT-infected plants showing leaf areas of 31.6–38.8% of the mock infected controls at day 16 (Fig. 6A). Comparable leaf area reductions were observed in the CDLR control mutants. However, leaf sizes were less affected in the PVY mutants with inserted CpG- and UpA-high sequences in R3, (areas of 64.4% and 67.8% of mock-infected leaves respectively; *p* = 0.02 and *p* = 0.009; Fig. 6B). Those with more extensive mutation over all three regions (R123-CpGH and R123-UpAH) showed actual increases in leaf size, similar to those of the mock-infected plants at 16 dpi and substantially greater than the WT-infected plants (p = 0.0004 and p = 0.0001).

**Figure 7:**
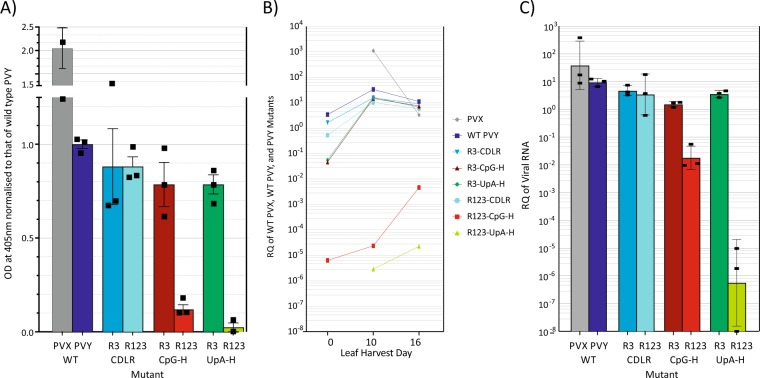
Quantification of PVY replication by protein ELISA and qPCR for viral RNA. **(A)** Quantification of viral capsid by DAS-ELISA in leaf lysates by ELISA Column at 10 dpi. Values were normalized to that of WT PVY. Bar heights show mean values of three replicates, error bars show standard deviations. (**B)** qPCR time-course quantitation of viral RNA at 07, 10, and 16 dpi, expressed as the ratio of viral RNA copies to those of the housekeeping gene, protein phosphatase 2A. (**C**) Quantitation of viral RNA by qPCR, expressed as the ratio of PYV RNA copies to those of the housekeeping gene, protein phosphatase 2A, at 10 dpi. Bar height show geometric mean ratios, error bars show standards deviation of log10 transformed data; square data-points indicate values for separate biological replicates.

Replicating virus was quantified in leaves collected from *N. benthamiana* agroinoculated with PVX, WT PVY and mutant viruses by double antibody sandwich ELISA (DAS-ELISA) using antibodies against the structural coat protein of PVY (Fig. 7A). PVY capsid could be detected at similar levels to WT for the CDLR mutants and the R3 CpGH and UpAH mutants, whereas the R123-CpGH and R123-UpAH viruses showed only 10% or undetectable levels compared to WT virus respectively. Detection of PVY by quantitative (real-time) PCR enabled detection over a greatly expanded quantitative range. Using this method, the similarity of replication levels of WT, R3-CDLR, R123-CDLR and R3-CpGH and R3-UpAH mutants was confirmed (values within 1–1.5 logs of WT) but the assay revealed >1000-fold reduction in replicating levels of the R123 CpG-H mutant, and a >10 million-fold reduction in plants infected with R123-UpAH.

### Antiviral RNAi does not restrict PVY mutants with elevated CpG/UpA frequency

*Nicotiana benthamiana* plants are deficient in RDR1 and additional knockdown (KD) of RDR6 prevents the mounting of a strong 21/22 nt siRNA mediated-PTGS response, leading to increased susceptibility to RNA virus infection^28,29^. To investigate whether this pathway was involved in the CpG or UpA-associated attenuation phenotypes, RDR6 knock down (KD) plants using a TRV-based VIGS vector were inoculated with R123-CpGH and R123-UpAH mutant viruses and replication compared with of the WT PYV. Effective KD of RDR6 to 20–40% of constitutive levels was verified by RT-PCR quantitation of mRNA sequences (Fig. S8; Suppl. Data).

**Figure 8:**
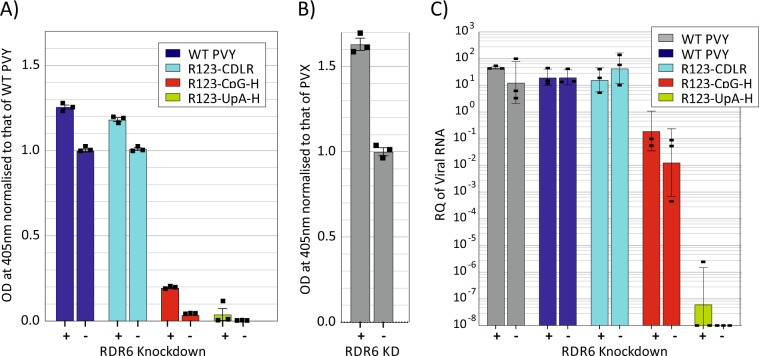
Impact of RDR6 knock down on PVY replication. (**A)** Quantitation of PYV viral capsid by DAS-ELISA in topmost third leaf at 10 dpi of *N. benthamiana* with knockdown of RDR6 (right-hand column) and of a GUS control (left-hand column). Plants were infected with WT PVY and compositionally altered mutants 9 days after knockdown. Values were normalized to that of WT PVY from GUS (control)- silenced *N. benthamiana*. (**B**) Comparative effect of RDR6 k/d of PVX replication as determined by DAS-ELISA detection of PVX viral particles. Values were normalized to that of PVX from GUS (control)- silenced *N. benthamiana*. Bar heights show mean values of three biological replicates, error bar show standard deviations. (**C**) Parallel evaluation of replication of PVY WT and mutant viruses by qPCR for PYV RNA. Column heights show geometric mean tire of three biological replicates; error shows show standard deviations of log-transformed values; square data-points indicate values for separate biological replicates.

Replication was quantified by ELISA (Fig. 8A), and by RT-PCR (Fig. 8C). The WT and CDLR mutants of PVY showed small increases in PVY replication in both ELISA and qPCR assays in the RDR6 KD plants compared to the GUS control, while effects of siRNA-mediated restriction were greater in the PVX control. Similarly to WT PVY, there was a limited effect of RDR6 KD on the replication of the R123-CpGH and R1-UpAH mutants that did not revert to WT PVY levels. These findings indicate that reduction in siRNA-mediated restriction of PVY has little or no effect on the severe attenuation of compositionally altered mutants of PVY.

## Discussion

This study documents profound attenuation of a plant RNA virus with modified dinucleotide compositions and reproduces previous observations for severe replication defects of mammalian RNA viruses and HIV-1 with elevated frequencies of CpG and, in echovirus 7 (E7), additionally of UpA dinucleotides^3–6^. The functional similarities of the underlying attenuation mechanisms between vertebrates and plants are a focus of this discussion.

### Host compositional mimicry in plant and vertebrate RNA virus genomes

Many groups of RNA viruses infecting eukaryotes recapitulate the suppression of CpG and UpA dinucleotides observed in host cell mRNA sequences (Fig. 2)^11,19,30^, as if they were either subject to the same compositional constraints that drove the evolution of cellular sequences or cells have evolved to sense non-self RNA based on RNA composition. Suppression of UpA was universal in all plant RNA viruses analysed (Fig. 2), with mean frequencies (expressed as observed/expected frequencies based on G + C content) of 0.68 (±0.08), overlapping with the range in *N. attenuata* mRNA (0.61 ± 0.09), both comparable in degree to mammalian RNA viruses^11^. The underlying basis for UpA suppression in plant mRNA sequences is unclear. The existence of universal although lesser degree of UpA suppression in host DNA sequences of plants and other eukaryotes suggests that it, at least part, originates from a mutagenic process occurring during DNA replication, since the vast majority of DNA sequences are not expressed and potentially fix mutations in the absence of compositional selection. Our previous modelling of dinucleotide frequencies in mammalian and other eukaryotic genomes indeed showed that UpA under-representation could arise secondarily from methylation-associated CpG depletion, rather than direct targeting of TpA dinucleotides in genomic DNA^11^. This mechanism potentially extends to plant genomes. However, such mutagenic effects are unlikely to be reproduced in the enzymatically quite different process of RNA-dependent RNA transcription of RNA virus genomes. The observation for universal suppression of UpA in RNA viruses therefore supports the hypothesis that they have adapted their composition for replication in the plant cell cytoplasm, very much as previously discussed for mammalian RNA viruses^3,5^.

There was substantial suppression of CpG in plant genomic DNA and mRNA sequences (Fig. 1) although not to the extent observed in equivalent subsets of mammalian genomic sequences. Methylation of the cytosine in CpG sites confers a greater likelihood of being copied as a T during DNA transcription leading to a general suppression of frequencies over longer evolutionary timescales. The lesser degree of suppression may be related to the lower degree of methylation of CpG sites in plant genomic DNA (approximately 25% of sites) compared to 75% in mammalian genomes^31^. Supporting this relationship, suppression of CHG frequencies was much more restricted (O/E ratio of 0.94), commensurate with methylation frequencies of 6.7%, while the absence of detectable suppression of CHH frequencies matched to its extremely low frequency (1.7%) and reversible nature of its methylation.

CpG frequencies in plant RNA viruses (and host genomes) showed both similarities and differences from the universal suppression of UpA. Firstly, not all RNA viruses showed suppression, and for those groups where it was observed, the degree of under-representation was related to G + C content of the genomes (Fig. S1; Suppl. Data). This pattern of CpG under-representation in the virus subset comprising picorna-like viruses, *Tymovirales*, luteo/tombus/sobemo and –strand RNA viruses reproduced the relationship between CpG representation and G + C content in +strand and −strand mammalian RNA viruses and small DNA viruses^11^. However members of several other plant virus ssRNA + families (*Beniviridae, Virgaviridae*, *Bromoviridae* and *Closteroviridae*) and dsRNA viruses (*Reoviridae*, *Partitiviridae*) showed little or no GpG suppression (Fig. 2; S1B, Suppl. Data), as previously observed in vertebrate dsRNA viruses^32^. In the case of dsRNA viruses, the nature of their genome precludes its exposure to the cytoplasm as dsRNA would be readily targeted by pattern recognition receptors (PRRs) such as RIG-I and toll-like receptor 3 (TLR3) in vertebrate cells, along with downstream interferon-stimulated effector pathways such as RNAseL. Virus genomic dsRNA may be similarly targeted by siRNA in plants. Under these circumstances, effective shielding of the genome may make suppression of CpG and UpA frequencies unnecessary although such viruses will still produce compositionally abnormal mRNAs that may be targeted by ZAP and RNAseL^4,5^. The lack of CpG suppression in the other virus groups is less readily explained - replication of members of the *Beniviridae, Closteroviridae, Virgaviridae* and *Bromoviridae* occurs in compartments separated from the cytoplasm^33–36^, but this is a general feature of plant RNA virus replication and occurs similarly in tombusviruses, tymoviruses and potyviruses^37–39^ where CpG frequencies are systematically suppressed.

The evidence of mimicry of host genome composition by PVY, other potyviruses and many other RNA plant viruses motivated us to mutate CpG and UpA frequencies to determine their effects on virus replicative fitness. This represents a first step towards uncovering the underlying pathways that modulate viral RNA composition in plants.

Increasing UpA frequencies produced a dramatic reduction in the replication ability of PVY. The >8 log reduction in viral loads of R123-UpAH PVY in *N. benthamiana* (Fig. 7) vastly exceeded the 1.2–3 log reduction in the infectivity of the human enterovirus, E7 with a similar degree of mutagenesis^3,5^. CpG-high mutants of PVY were comparatively less attenuated, with approximately 1,000-fold reduction in replication, in this case around 1–2 logs less than observed in similarly mutated mutants of E7. A role of dinucleotide frequency changes in the attenuated phenotypes was supported through the use of CDLR controls, in which sequences were extensively mutated but retained native dinucleotide frequencies and coding. These showed equivalent replication ability to WT PVY, demonstrating that the attenuation of UpA and CpG-high mutants was not the result of disruption of undocumented RNA structure-based replication elements in the genome or undocumented alternative reading frames with functional roles on virus replication.

How the plant recognizes and responds to these compositionally modified sequences is currently unclear. The attenuation of mammalian RNA viruses has been recently shown to be mediated through the action of a novel restriction pathway, in which direct binding of ZAP to CpG-enriched viral RNA sequences somehow induces a profound curtailment of its replicative ability. We have recently shown that ZAP similarly directly binds to and restricts the replication of UpA-enriched mutants of E7 viruses and replicons^21^ although whether this occurs through a shared, polyvalent binding site or through alternative binding domains in ZAP awaits structural studies. We have also obtained evidence for the involvement of RNAseL–mediated restriction of CpG-high E7 mutant replication, signalled through activation by oligoadenylate synthetase 3 (OAS3)^21^, a PRR that to date has been considered to specifically target dsRNA. The unexpected involvement of these pathways and their independence from conventional IFN-mediated antiviral responses^3^ further exemplify the complexity of virus/host interactions, even at the single cell level. The possibilities that these restriction pathways are shared in plant is explored in the following discussion.

At its broadest, plant virus immunity can be divided into two principal strategies. siRNA-mediated cleavage and decay of viral RNA sequences represents a potent defence mechanism shared across eukaryotes and considered by many to represent the primary defence of plants against RNA virus infection^40^. This conjecture is supported by the almost universal development by plant viruses of one or more anti-siRNA evasion pathways, typically through encoding proteins termed viral suppressors of RNA silencing (VSRs) (reviewed in^41^). In the case of potyviruses, two different VSRs have been identified, HC-Pro that acts through a variety of translational, RNA binding and exosome inhibition pathways (reviewed in^42^) and VpG that inhibits expression of suppressor of gene silencing 3 (SGS3) a key component of the siRNA pathway^43^. The difference in replication levels of WT PYV in RDR6 KD plants and controls (Fig. 8) demonstrated a degree of virus control by the siRNA pathway and that the pathway was active in the PYV/*N. benthamiana* model. However, the lack of phenotypic reversion of the CpG- and UpA-high PYV mutants indicates that RNAi (PTGS) is unlikely to play a role in their attenuation. This conclusion is mechanistically supported by the nature of the dsRNA motifs targeted by siRNA. There is no current indication in other systems that increased frequencies of CpG or UpA dinucleotides would enhance the activity of this pathway.

The second and more extensive component of plant defence involves PAMP-triggered immunity (PTI) and effector-triggered immunity (ETI). During PTI an array of predominantly cell surface expressed pathogen recognition receptors (PRRs), consisting of receptor kinases and receptor-like proteins, detect a vast range of different PAMPs, expressed on the surfaces of bacteria and fungi in the apoplastic space of plant cells^44^. These dimerize and signal through SERKs and SOBIR1 to activate a large number of anti-microbial responses. Only few cases have been reported on the (extracellular) sensing of plant viruses by PRRs. In contrast, most plant viruses trigger an ETI after being sensed by intracellular sensors of innate immunity, the biggest class represent nucleotide binding-leucine-rich repeat (NLR) proteins encoded by single dominant resistance (*R*) genes^45^. Their triggering by viral effectors mostly comes with a hypersensitive response (HR), observed by the formation of necrotic local lesions and resulting from apoptosis, a programmed cell death response. NLRs are also present in animals and structurally similar to those of plants. Despite many structural and functional similarities between these and in the stress response pathway conserved across eukaryotes, PRRs, its downstream signalling mechanisms and antimicrobial and R/NLR protein responses represent a system that may have evolved independently and convergently in each kingdom^46–52^. In light of this it remains to be questioned whether there are direct homologues of mammalian ZAP or RNaseL in plant cells that mediate the restriction of PVY mutants in the current study.

ZAP is a member of the poly-adenosine diphosphate ribosyl transferase protein (PARP) family that is widely distributed across all eukaryotes^53^. While PARPs typically play roles in DNA and RNA metabolism and repair and show a predominantly nuclear cellular distribution, indirect genetic evidence for positive selection suggests that PARP4, PARP9, PARP14 and PARP15 may also have been co-opted to function in vertebrate innate immunity^54^. Plants possess three paralogues of PARP; however, all three show predominantly nuclear distributions and lack the typical zinc finger domains associated with RNA binding and cleavage; they consequently appear unlikely candidates for functional homologues of ZAP for CpG and UpA recognition in plants. In the absence of any clear candidate pathways for CpG or UpA-enriched RNA sequences, a more robust strategy to identify recognition proteins is to exploit their potential binding to immobilized RNA targets of different compositions (as used to detect ZAP binding to UpA- and CpG-high E7 sequences^21^) and to identify and characterize bound proteins by mass spectrometry. A comparative analysis of high UpA- and CpG-high RNAs in mammalian, arthropod (mosquito, tick) and plant cytoplasmic preparations is currently planned.

## Materials and Methods

### Plant material and bacterial strains and growth conditions

*Nicotiana benthamiana* plants were grown in contained room at 23 °C ± 2 °C in a 16/8 h light and dark cycle. The *Agrobacterium tumefaciens* strain GV3101 was used for agroinfiltration of PVY cDNA constructs; strain LBA4404, was used For TRV based VIGS constructs. Both strains of *Agrobacterium* were grown at 28 °C shaking at 200 rpm.

### Clone construction

Clone N19N21 pCAMBIA PVY N605 GFPCP was provided by Elisabeth Johansen^55^ comprising the sequence of PYV isolate N605 cloned into the pCAMBIA binary vector (Marker Gene Technologies). Four introns were introduced to facilitate propagation in *Escherichia coli* and *Agrobacterium tumefaciens*. The inserted introns were the modified second intron of the *Solanum tuberosum* light-inducible tissue-specific gene^56^ and the second intron of *Phaseolus vulgaris* nitrite reductase gene^57^. The full sequence of the clone and regions of native and introduced (intronic) genome sections are provided as a Supplementary File.

For mutant construction, a PVY sub-clone segment, between *Bst*XI and *Hpa*I sites was sub-cloned from wtPVY-pCambia into pJET (ThermoFisher Scientific) using PCR cloning giving wtPVY-pJET construct. Mutant PVY insert sequences (GeneArt; Life Technologies listed in Supplementary Data) were first cloned into wtPVY-pJET using sites *Nco*I and *Bgl*II for Regions 3 and sites *Bst*XI and *Nco*I for Regions 1 and 2 and then introduced into wtPVY-pCambia giving six compositionally modified PVY-pCambia constructs (Table 1).

### Screening for and mutation-introduced intron like splice sites

Mutated PVY regions were screened using NetGene2 Server^58^ for mutation-introduced intron splice sites. Sites with confidence scores higher than 0.8 were reverted back to WT sequence.

### *Agrobacterium* mediated infection

For agroinfiltration of *N. benthamiana* with PVY cDNA, first, *A. tumefaciens* was electroporated (at 25 mF, 2.5 kV and 200 ohms using Bio-Rad Gene Pulser (Bio-Rad) with the respective PVY.pCambia vector, and a single colony was grown overnight in LB3 medium (10 g/L tryptone, 5 g/L yeast extract, 4 g/L NaCl, 1 g/L KCl, 3 g/L MgSO4.7H2O) supplemented with 100 μg/ml kanamycin and 100 μg/ml rifampicin. From the overnight culture, 600 μL were inoculated into 3 ml of induction medium (10.5 g/L K_2_HPO_8_, 4.5 g/L KH_2_PO_8_, 1 g/L (NH_4_)_2_SO_4_, 0.5 g/L sodium citrate dihydrate, 0.25 g/L MgSO_4_, 0.2% (w/v) glucose, 0.5% (v/v) glycerol, 50 mM acetosyringone and 10 mM MES) and incubated overnight at 28 °C shaking at 200 rpm. The next day, broth was centrifuged at 3000 rpm for 15 min at 28 °C. Following decanting of supernatant, the pellet was resuspended in infiltration medium (30 g/L sucrose, 4 g/L Murashige and Skoog medium, 50 mM acetosyringone and 10 mM MES) and diluted to an optical density at 600 nm (OD_600_) of 0.4. Plant leaves were agroinfiltrated at the basal side of three weeks old *N. benthamiana* leaves^59^.

Gene knockdown in *N. benthamiana* was performed by virus induced gene silencing (VIGS) using the tobacco rattle virus (TRV) vector system. To this end, *N. benthamiana* was agroinfiltrated with a combination of TRV1 and TRV2, containing a sequence of the target gene to be silenced. For RDR6 silencing, *A. tumefaciens* was transformed independently with the either TRV1 or TRV2-RDR6 plasmids, after which, a single colony of each transformant was grown overnight in LB3 medium as described above. Similarly, overnight broth cultures were introduced into induction medium for overnight incubation, then centrifuged and resuspended in the infiltration medium. Both infiltration medium preparations, one for *Agrobacterium* harbouring TRV1 and another for *Agrobacterium* harbouring TRV2-RDR6, were diluted to OD_600_ of 0.4. Equal volumes from both preparations were then mixed together and agroinfiltrated into the basal side of two weeks old *N. benthamiana* leaves^60^. Nine days after VIGS agroinoculation, plants were agroinoculated with the PVY mutants. To verify on the onset of gene silencing, as a positive control, plants were infiltrated with TRV-PDS and monitored on bleaching of chlorophyll.

### Measuring the upper most four leaves surface area

The four most top leaves of agroinoculated *N. benthamiana* were harvested and placed flat against white background in proximity to a linear measuring ruler. Pictures were taken using Canon SLR camera (Cannon) and leaf area was measured using ImageJ software. Differences in leaf area were statistically evaluated using GraphPad Prism software.

### Detecting fluorescence

3 × 3 mm sections of topmost third leaf from agroinoculated *N. benthamiana* were screened for green fluorescence signal using ZEISS Observer. Z1 fluorescence microscope mounted with Filter Set 38 for excitation and emission wavelengths of 470/40 and 525/50 respectively. Pictures were processed using AxioVision Release 4.9.1 software.

### Virus extraction and quantification using enzyme linked immunosorbent assay (ELISA)

Virus was extracted from *N. benthamiana* leaf tissue using extraction buffer (PBS, 2% (v/v) Tween 20, 2% polyvinyl pyrrolidone (PVP) (Sigma), pH 7.4) using TissueLyser II (Qiagen). Double antibody sandwich (DAS) ELISA using 96-well flat bottom ELISA plates was designed to quantify virus titre. For PVY detection, wells were coated with capture antibody, anti-PVY coating Ab (Prime Diagnostics, Netherlands), diluted in bicarbonate buffer (1.59 g/L Na_2_CO_3_, 2.94 g/L NaHCO_3_, pH 9.6) and washed with 2% PBS-T pH 7.4. Extracted virus samples were further diluted in extraction buffer (20-fold dilution) before being added to the wells and were incubated at 4 °C overnight. Detection was with anti-PVY alkaline phosphate conjugated antibody (Prime Diagnostics, Netherlands) diluted in extraction buffer and incubated at 4 °C overnight. For PVX detection, same procedure was followed however anti-PVX coating Ab (Prime Diagnostics, Netherlands) and anti-PVX alkaline phosphate conjugated antibody (Prime Diagnostics, Netherlands) were used. Following overnight incubation, plates were washed with 2% PBS-T pH 7.4, and *p*-nitrophenylphosphate (pNPP) (Sigma) was used as enzyme substrate. Colour was allowed to develop for 30 minutes at room temperature before the addition of 3 N NaOH to stop the reaction. Absorbance was read at 405 nm.

### RNA extraction and *DNase* treatment

Total RNA was extracted from 100 mg *N. benthamiana* leaf tissue using TRI Reagent Solution (Invitrogen) homogenized using TissueLyser II (Qiagen). Following homogenization, chlorophorm was added (200 ul/1 ml of TRI reagent) and centrifuged at 12,000 g for 20 minutes at 4 °C, after which, the aqueous phase was transformed into equivolume of isopropanol, and centrifuged at 12,000 g for 20 minutes at 4 °C. Pellet RNA was washed 3x with 75% ethanol before being resuspended in DNase/RNAse-free distilled H_2_O. Extracted RNA was treated with RQ1 *Dnase* (Promega) at 37 °C for 1 hour.

### Confirming RDR6 knockdown

Total RNA extracted from *N. benthamiana* agroinoculated with VIGS-silenced on RDR6, or GUS (negative control) and treated with RQ1 *Dnase* (Promega), was used as a template for reverse transcribing cDNA using SuperScript™ II Reverse Transcriptase (ThermoFisher Scientific) and RDR6_cR primer. Following cDNA synthesis, primers pair RDR6_F and RDR_R were used to PCR amplify the cDNA strand using *Taq* 2X Master Mix (NEB). PCR products were run on 1.0% agarose gels to confirm absence of RDR6 representing band.

### Relative quantification using RT-qPCR

The qPCR was done using TaqMan qPCR Master Mix (ThermoFisher Scientific) on a OneStep Plus Real-Time PCR system (ThermoFisher Scientific). The primer pair PVY_ F and PVY_R and the probe PVY_P are listed in Table S1 (Suppl. Data) was designed to anneal to the coat protein of the viral genome as described previously^61^. The data produced during qPCR was processed using StepOne Plus software (ThermoFisher Scientific). Data was presented as relative quantification (RQ) using phosphatase 2 A gene, *PP2A*, as endogenous reference^62^. RT-qPCR conditions were 30 min incubation at 48 °C, followed by 10 min at 95 °C, then 40 cycles of 1 min at 60 °C plus 15 s at 95 °C.

### Sequence bioinformatics analysis

Plant virus sequences datasets was constructed from representative sequences of each plant virus species were obtained from the ICTV Virus Metadata Resource^63^; n = 1193; *Amalgaviridae*: n = 6; *Chrysoviridae*: n = 8; *Partitiviridae*: n = 46; *Reoviridae*: n = 111; *Caulimnoviridae*: n = 178, Other *Ortervirales* (*Metaviridae, Pseudoviridae*): n = 51; Bunya-like viruses (*Fimoviridae, Phenuiviridae, Tospoviridae*): n = 44; *Rhabdoviridae*: n = 108; *Secoviridae*: n = 125; *Betaflexiviridae*: n = 268; *Potyviridae*: n = 202; *Alphaflexiviridae*: n = 182; Other tymovirales (*Deltaflexiviridae, Gammaflexiviridae*): n = 11; *Tymoviridae*: n = 85; *Sobemovirus*: n = 18; *Benyviridae*: n = 22; *Luteoviridae*: n = 157; *Virgaviridae*: n = 247; *Bromoviridae*: N = 152; *Chlosteroviridae*: n = 337). Sequences were split into annotated genes to enable analysis of coding regions only. Families and other taxonomic groups representing Baltimore Groups 3–5 and 7 were analysed.

DNA genome sequences of *Arabidopsis thaliana* (chromosomes 1–5), *Zea mays* (chromosome 9) and *Nicotiana attenuata* (chromosome 1) were retrieved from Refseq and divided into 5000 base fragments for dinucleotide composition analysis. Coding region sequences from mRNA sequences of the three plant species and *N. tomentosiformis* were similarly downloaded from the Refseq database and subsequently filtered to remove redundant sequences by selecting the longest splice variant of each mRNA. From these, sequences longer than 450 bases were analysed for dinucleotide composition.

Mono- and dinucleotide composition measurements were performed in SSE version 1.3 (Simmonds, 2012). Observed/expected frequencies of CpG and TpA/UpA dinucleotides were calculated by normalisation for G and C mononucleotide content. Normalisation of CHG and CHH frequencies was performed by correction for frequencies of their two component dinucleotides instead of the three mononucleotides as the former may themselves by substantially over- or underrepresented. Normalised frequencies of CHG and CHH were calculated as:

$$\begin{array}{rcl}{\rm{nf}}({\rm{CpHpG}}) & = & {\rm{f}}({\rm{CpGpH}})\ast ({\rm{f}}({\rm{A}})+{\rm{f}}({\rm{C}})+{\rm{f}}({\rm{U}}))/(({\rm{f}}({\rm{CpA}})+{\rm{f}}({\rm{CpC}})\\  &  & +\,{\rm{f}}({\rm{CpU}}))\ast ({\rm{f}}({\rm{ApG}})+{\rm{f}}({\rm{CpG}})+{\rm{f}}({\rm{UpG}})))\\ \mathrm{nf}(\mathrm{CpHpH}) & = & (({\rm{f}}({\rm{CpGpH}})\ast ({\rm{f}}({\rm{A}})+{\rm{f}}({\rm{C}})+{\rm{f}}({\rm{U}}))/(({\rm{f}}(\mathrm{CpA})+{\rm{f}}(\mathrm{CpC})\\  &  & +\,{\rm{f}}({\rm{CpU}}))\ast ({\rm{f}}({\rm{ApA}})+{\rm{f}}({\rm{CpA}})+{\rm{f}}({\rm{UpA}}))))+({\rm{f}}({\rm{CpGpH}})\\  &  & \ast \,({\rm{f}}({\rm{A}})+{\rm{f}}({\rm{C}})+{\rm{f}}({\rm{U}}))/(({\rm{f}}({\rm{CpA}})+{\rm{f}}({\rm{CpC}})+{\rm{f}}({\rm{CpU}}))\\  &  & \ast \,({\rm{f}}(\mathrm{ApC})+{\rm{f}}(\mathrm{CpC})+\,({\rm{UpC}}))))+(\,({\rm{CpGpH}})\ast ({\rm{f}}({\rm{A}})\\  &  & +\,{\rm{f}}({\rm{C}})+{\rm{f}}({\rm{U}}))/(({\rm{f}}({\rm{CpA}})+{\rm{f}}({\rm{CpC}})+{\rm{f}}({\rm{CpU}}))\ast ({\rm{f}}({\rm{ApU}})\\  &  & +\,{\rm{f}}({\rm{CpU}})+{\rm{f}}({\rm{UpU}})))))/3\end{array}$$

Analyses of suppression of synonymous variability (SSV) and secondary structure predictions for PVY by calculation of minimum folding energy differences (MFED) was performed using SSE as previously described^64^. Analyses were made using all (near) complete genome sequences of PVY available on Genbank in November, 2014. Sequences <1% divergent from others were discarded. Accession numbers for the analysed sequences are provided in Table S2 (Suppl. Data).

## Supplementary information

Dataset 1

## Data Availability

Most data generated or analysed during this study are included in this published article (and its Supplementary Information files). Any other datasets generated during and/or analysed during the current study are available from the corresponding author on reasonable request.
