# Supplementary material for: A functional investigation of the suppression of CpG and UpA dinucleotide frequencies in plant RNA virus genomes
Source: Sci Rep. 2019 Dec 4;9:18359. doi: 10.1038/s41598-019-54853-0 (PMC6892864; doi:10.1038/s41598-019-54853-0)
Supplement: Supplementary file 1 — Dataset 1 [file 41598_2019_54853_MOESM1_ESM.docx]

SUPPLEMENTARY DATA

For

A functional investigation of the suppression of CpG and UpA

dinucleotide frequencies in plant RNA virus genomes

**Ahmad Ibrahim^1^, Jelke Fros^1,2^, Andre Bertran^2^, Ferdyansyah Sechan^1^,**

**Valerie Odon^1^, Leslie Torrance^3^, Richard Kormelink^2^, Peter Simmonds^1*^**

Table S1. List of primers and probes used.

| **Label** |  | **Sequence** | **Description** |
| --- | --- | --- | --- |
| PVY_F | Forward | ACATAGGAGAAACTGAAATGCCAAC | Binds to CP of PVY for RT-qPCR assay |
| PVY_R | Reverse | GTTGATGTTTGGCGAGGTTCCA |  |
| PVY_P | Probe | GTGATGAATGGGCTTATGGTTTGGTGCATTG |  |
| PVX_F | Forward | AAGCCTGAGCACAAATTCGC | Binds to CP of PVX for RT-qPCR assay |
| PVX_R | Reverse | TTCAGCTTCAGACGGTGGC |  |
| PVX_P | Probe | AATGGAGTCACCAACCCAGCTGCC |  |
| P2P_F | Forward | ACCCTGATGTTGATGTTCGC | Binds to endogenous *PP2A* gene used as a reference gene in RT-qPCR assays |
| P2P_R | Reverse | AGGGATTTGAAGAGAGATTTCTAGTACTCT |  |
| P2P_P | Probe | ACTTCAGTCTATTGATAACGTCATGATGTCAGGCT |  |
| RDR_cR | Reverse | AGCTAGGACCATTGCGAGG | Used for reverse transcription of RDR6 mRNA |
| RDR6_F | Forward | CCCTATCGTCTGAATCAGCGG | Used for PCR amplification of RDR6 cDNA |
| RDR6_R | Reverse | CCTACCAATGGCTCCACTACCTG |  |
| pJET_F | Forward | GGGCCACTCCAGTGGAAATCGATGTTCTTCTTTTATTCT | Used to introduce PVY subclone into pJET cloning vector. |
| pJET_R | Reverse | GGGGGTGAGGTCACCGTTAACGCGGCCGCTCTCCCTATAGT |  |

Table S2. Sequence of PYV used for bioinformatic analysis.

O-Group: DQ309028, AF463399, HQ912864, FJ214726, HQ912865, AJ585196, HQ912914, PVU09509, HM367076, AB714134, HQ912873, JQ924285, EF558545, HQ912912, JX424837, KF770835, AB711154, HQ912891, AB711150, X12456, AJ585195, AB711149, AF237963, AB711155, AJ439544, EU563512, AB711151, AB711148

NTN group: X97895, KC634005, AJ890346, JN936416, HG810951, KC296440, HG810952, JQ969036, JN936433, AB461454, AJ889867, AJ890348, AJ890343, JN936439, AJ439545, HG810949, JN936426, JQ969037, JQ969035, KJ634023, AM113988, JF927755, JN083841, JN034046, AJ890349, JF927762, JN936419, AY745492, KJ801915, JQ969040, HM991454, JN936435, DQ157180, HQ912867, KC296436, KJ634024, JN083842, JQ971975, AB714135, AY166867, KC296432, EU182576, AB331518, AY884984, JN936422.

FIGURE S1


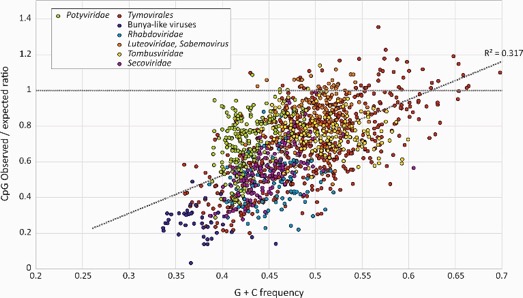
A) *Secoviridae*, *Potyviridae, Tymovirales*, luteo/tombus/sobemo, ambisense and –strand RNA viruses


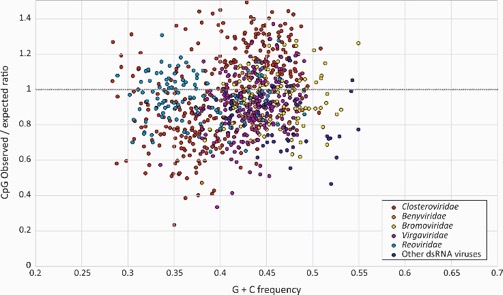
B) dsRNA RNA viruses, *Closteroviridae, Benyviridae, Bromoviridae* and *Virgaviridae*

Comparison of CpG and UpA frequencies of potyviruses with those of coding regions of individual genes (>450 bases) or polyproteins of the major plant virus families / groups.

FIGURE S2

Frequencies of CHG and CHH methylation-associated motifs in plant RNA viruses


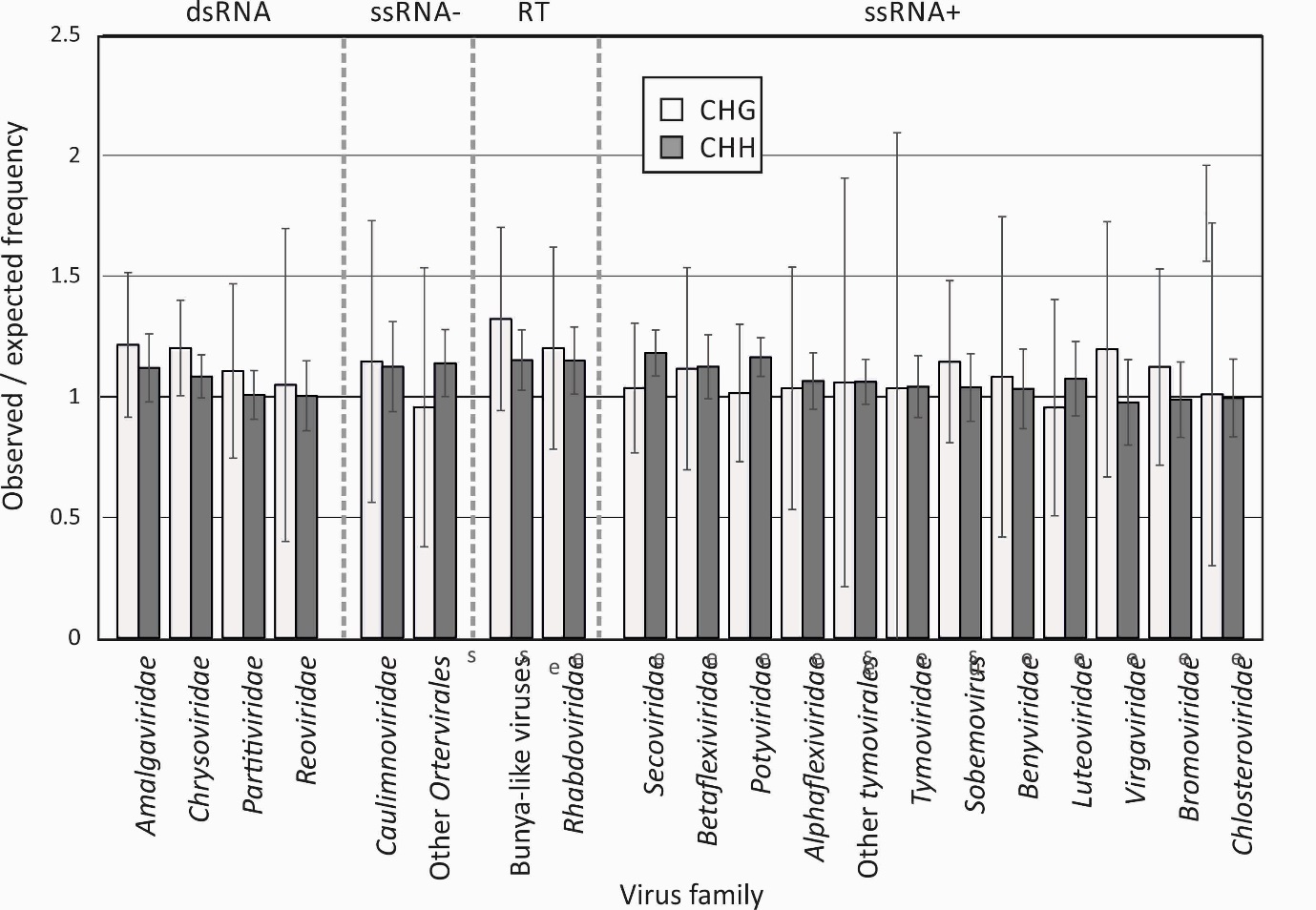


Observed to expected (O/E) ratios of CHG and CHH motifs in plant TNA virus genes (sequence lengths > 450 bases) divided into separate groups and families. These comprise dsRNA RNA viruses (Baltimore group 3), negative-stranded RNA viruses (ssRNA-; Group 5), reverse transcribing viruses (RT; Group 7) and plus-stranded RNA viruses (ssRNA+; Group 4). An O/E ratio of 1.0 (heavy line) is the expected frequency for sequences showing unbiased frequencies of each motif normalised to their constituent dinucleotide frequencies (see Methods). Bar heights show mean values; error bars show ±1 standard deviation. Standard deviations are relatively high for the CHG motif in view of its infrequency in relatively short RNA virus genomes.

FIGURE S3

Frequencies of CHG and CHH methylation-associated motifs in *N. attenuata* and PYV


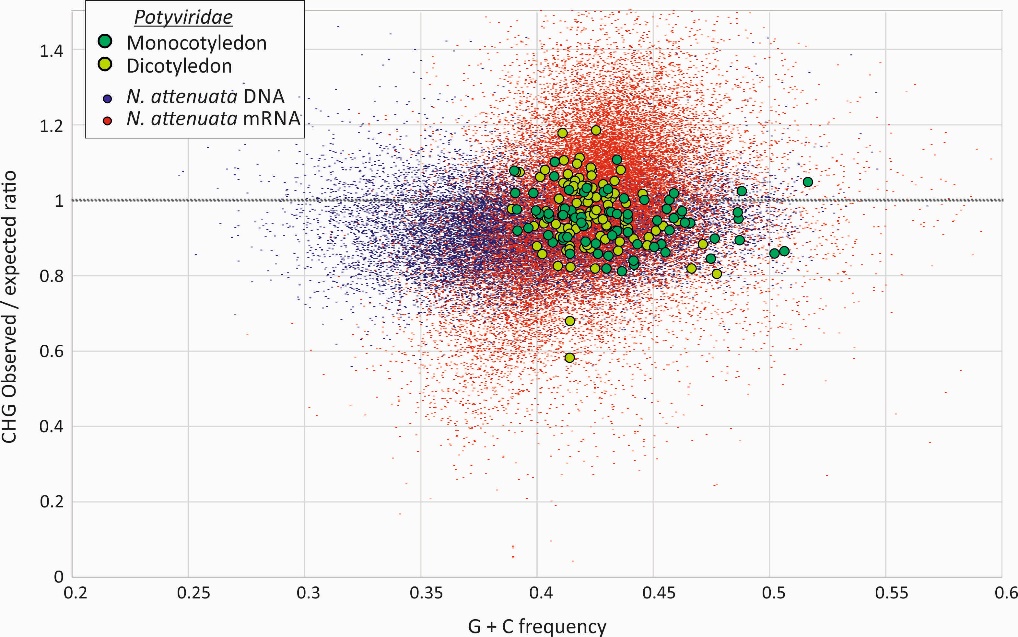


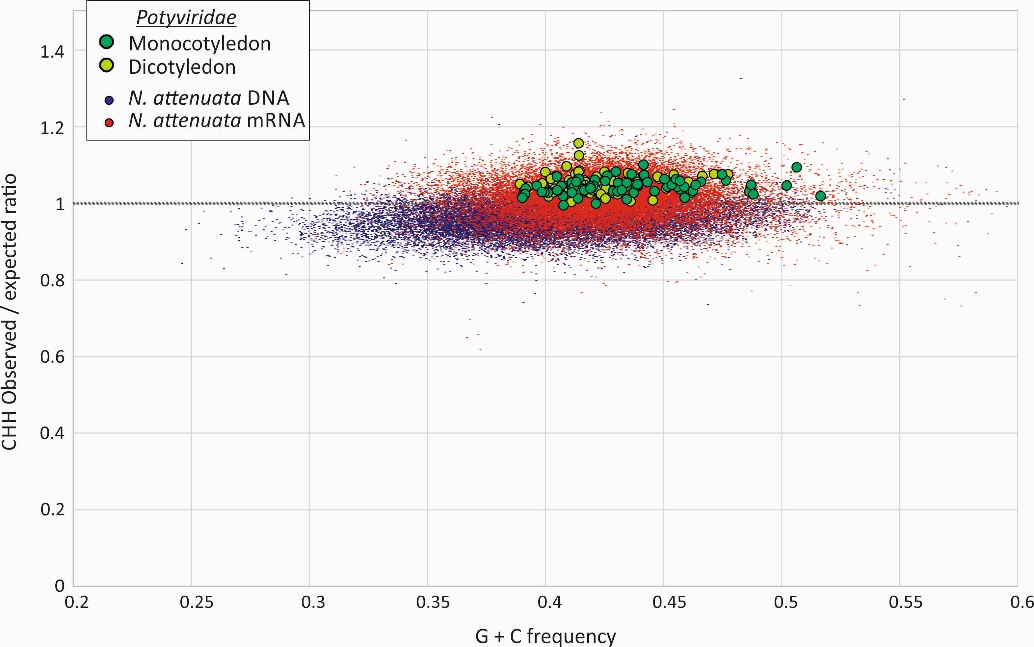


Frequencies of CHG (Cp(A,C,T)pG) and CHH (Cp(A,C,T)p(A,C,T)) motifs in *Nicotinia attenuata*  genomic DNA (in fragments 5000 bps), coding regions of mRNAs (>450 bases) and polyprotein genes of each potyvirus species (>450 bases) divided by host. The O/E ratio of 1.0 (heavy line) is the expected frequency of the two motifs based their mononucleotide and dinucleotide compositions (grey dotted line; see Methods).

FIGURE S4

CpG and UpA suppression in ordinary and necrotic (PVY^NTN^/PVY^N-Wi^) strains of PYV


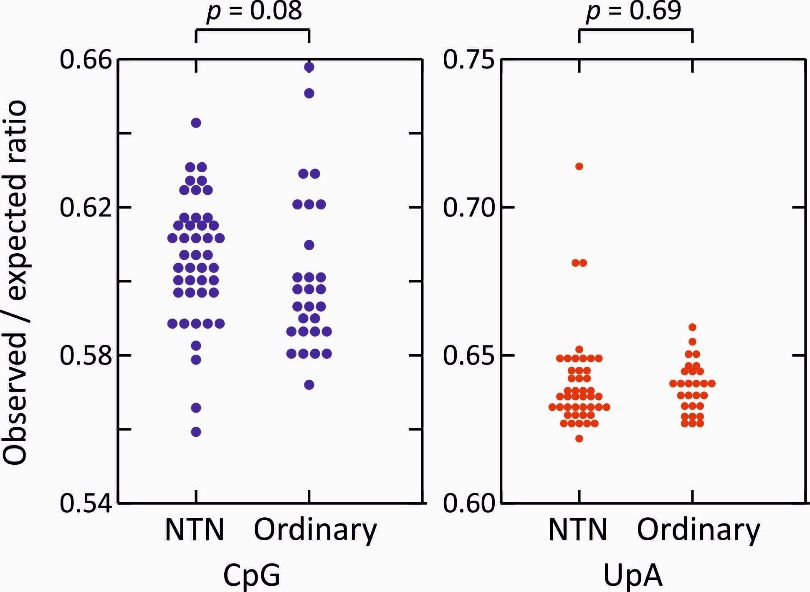


Observed to expected frequencies in the coding regions of PYV strains with different pathology phenotypes. The analysis was based upon previously characterised and sequenced ordinary (n=28) and necrotic (n=44) PYV isolates as listed in Methods. Values between groups were compared using the Kruskall-Wallace non-parametric test.

FIGURE S5

Genome organization of PVX, PVY and PVY clones and mutagenesis strategy

|  |
| --- |

Diagram showing genomic organization of PVY^NTN^ and the regions with altered CpG and UpA frequency. (A) Genomic organization of PVY as previously described [96] showing the sites of *Bst*XI and *Hpa*I between which the sub-clone region was used as during cloning of PVY mutants and the GFP gene upstream of the CP gene. (B) PVY with a single mutated region; Region 3 between *Nco*I and *Bgl*II sites. (C) PVY with three mutated regions; Region 1 between *Bst*XI and *Xho*I sites, Region 2 between *Xho*I and *Nco*I sites, Region 3 between *Nco*I and *Bgl*II sites.

FIGURE S6

| 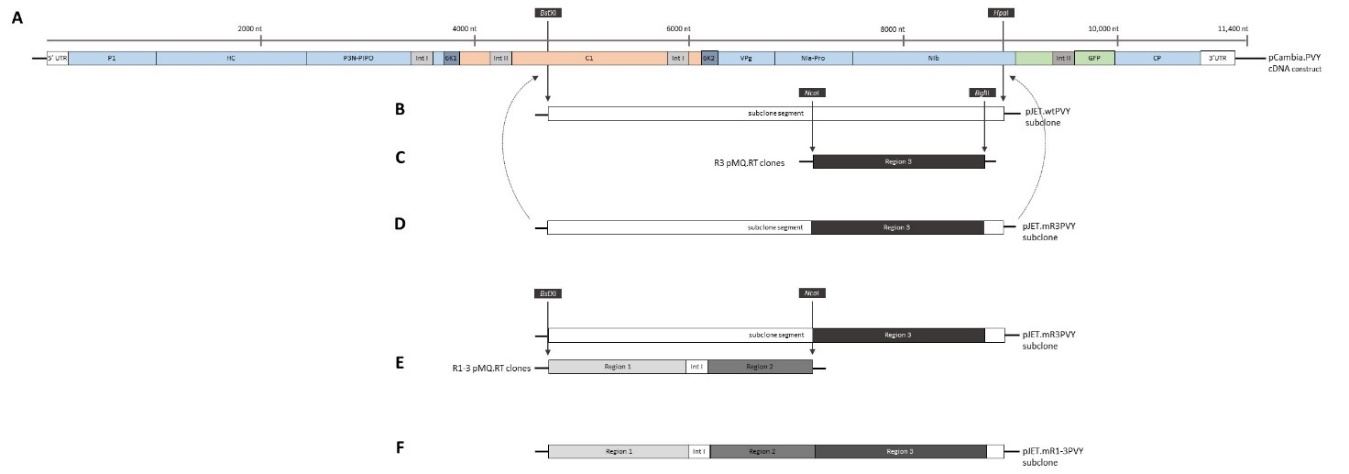 |
| --- |

Genomic organization of PVY^NTN^ and the regions with altered CpG and UpA frequency. (A) Genomic organization of PVY^NTN^ strain N19N21 with the four introduced introns, and the green fluorescence protein gene as part of pCambia vector. Between *Bst*XI and *Hpa*I restriction sites is the sub-clone fragment which was used during cloning of PVY mutants. (B) PVY sub-clone fragment introduced into pJET plasmid. (C) Region 3 with either altered CpG, UpA, or CDLR as received from GeneArt as part of pMQ plasmids. (D) PVY sub-clone fragment with Region 3 only having altered CpG or UpA frequencies, or CDLR, as part of pJET plasmid, before being reintroduced into PVY.pCambia. (E) Regions 1 and 2 with either altered CpG or UpA frequencies, or CDLR as received from GeneArt as part of pMQ plasmids. (F) PVY sub-clone fragment with Regions 1, 2, and 3 having altered CpG or UpA frequencies, or CDLR, as part of pJET plasmid, before being reintroduced into PVY.pCambia.

Figure S7

*N. benthamiana* showing systemic spread of PVX and PVY under UV light

| A | B | C |
| --- | --- | --- |
| **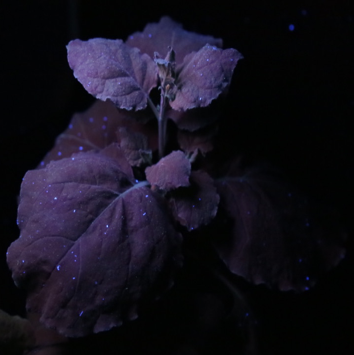** | **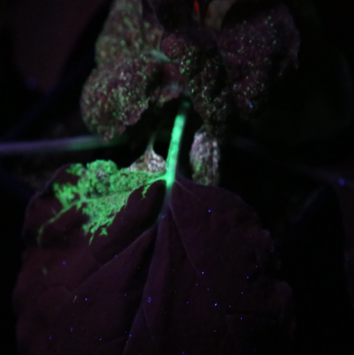** | **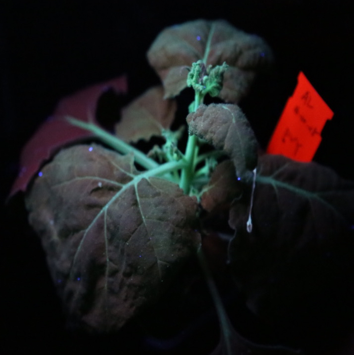** |

**A.** *N. benthamiana* agroinoculated with blank agrobacterium showing purple coloured cholorophyll autofluorescence. **B.** *N. benthamiana* agroinoculated with PVX showing spots of green fluorescnece. **C.** *N. benthamiana* agroinoculated with WT PVY showing diffused green fluorescence. All pictures were taken 16dpi.

Figure S8

Quantitation of knockdow on RDR6 and GUS by siRNA pre-treatment


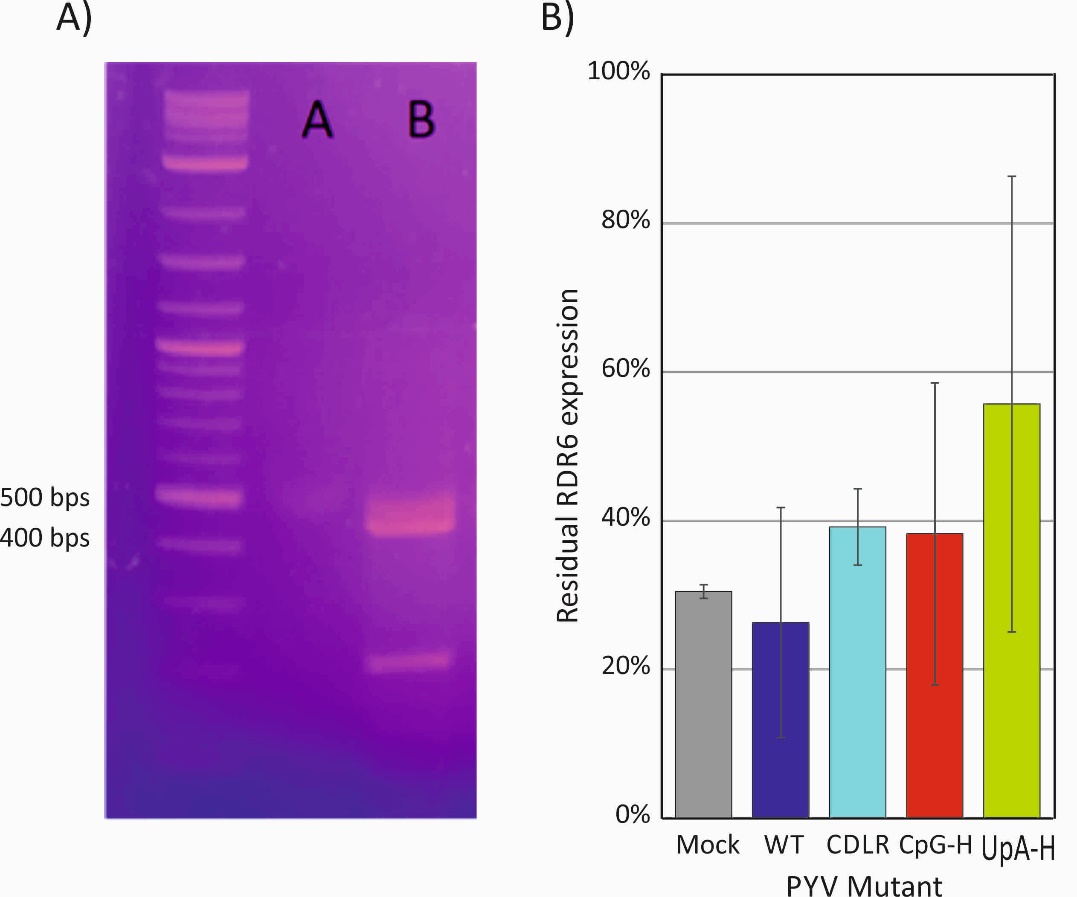


**A.** Amplicons from RT-PCR for RDR6 mRNA in cells with VIGS (lane A) and GUS (lane B; KD control) KD in *N. benthamiana* plants. **B**. qPCR of RDR65 mRNA sequences in RDR6 KD plants normalised to a housekeeping gene mRNA **(**phosphatase 2A) and then further normalised to similarly calculated RDR6 expression in the GUS (control) KD treated plants. Bar heights represent mean values of relative expression of RDR6 in KD and control plants in two biological replicates; error bars show SEMs.
